# Lactic Acid Fermentation of Cactus Cladodes (*Opuntia ficus-indica* L.) Generates Flavonoid Derivatives with Antioxidant and Anti-Inflammatory Properties

**DOI:** 10.1371/journal.pone.0152575

**Published:** 2016-03-29

**Authors:** Pasquale Filannino, Ivana Cavoski, Nadia Thlien, Olimpia Vincentini, Maria De Angelis, Marco Silano, Marco Gobbetti, Raffaella Di Cagno

**Affiliations:** 1 Department of Soil, Plant and Food Science, University of Bari Aldo Moro, Bari, Italy; 2 CIHEAM-MAIB, Mediterranean Agronomic Institute of Bari, Valenzano, Bari, Italy; 3 Unit of Human Nutrition and Health, Department of Veterinary Public Health and Food Safety, Istituto Superiore di Sanità, Roma, Italy; Indian Institute of Integrative Medicine, INDIA

## Abstract

Cactus pear (*Opuntia ficus-indica* L.) is widely distributed in the arid and semi-arid regions throughout the world. In the last decades, the interest towards vegetative crop increased, and cladodes are exploited for nutraceutical and health-promoting properties. This study aimed at investigating the capacity of selected lactic acid bacteria to increase the antioxidant and anti-inflammatory properties of cactus cladodes pulp, with the perspective of producing a functional ingredient, dietary supplement or pharmaceutical preparation. Preliminarily, the antioxidant activity was determined through *in vitro* assays. Further, it was confirmed through *ex vivo* analysis on intestinal Caco-2/TC7 cells, and the profile of flavonoids was characterized. Cactus cladode pulp was fermented with lactic acid bacteria, which were previously selected from plant materials. Chemically acidified suspension, without bacterial inoculum and incubated under the same conditions, was used as the control. *Lactobacillus plantarum* CIL6, POM1 and 1MR20, *Lactobacillus brevis* POM2 and POM4, *Lactobacillus rossiae* 2LC8 and *Pediococcus pentosaceus* CILSWE5 were the best growing strains. Fermentation of cladode pulp with *L*. *brevis* POM2 and POM4 allowed the highest concentration of γ-amino butyric acid. Lactic acid fermentation had preservative effects (P<0.05) on the levels of vitamin C and carotenoids. Two flavonoid derivatives (kaemferol and isorhamnetin) were identified in the ethyl acetate extracts, which were considered to be the major compounds responsible for the increased radical scavenging activity. After inducing oxidative stress by IL-1β, the increased antioxidant activity (P<0.05) of fermented cladode pulp was confirmed using Caco-2/TC7 cells. Fermented cladode pulp had also immune-modulatory effects towards Caco-2 cells. Compared to the control, fermented cladode pulp exhibited a significantly (P<0.05) higher inhibition of IL-8, TNFα and prostaglandins PGE2 synthesis. The highest functional effect was found using ethyl acetate extracts. In conclusion, fermentation, especially with *L*. *plantarum* strains and *L*. *brevis* POM4, enhanced the antioxidant and immune-modulation features of cladode pulp.

## Introduction

Cactus (*Opuntia* spp.), belonging to *Cactaceae* family, is cultivated in both hemispheres and all continents. Among all the species, *Opuntia ficus-indica* L. (known as cactus pear) is the most common. Native to Mexico, it is widely distributed and adapted to the arid and semi-arid regions of South and Central America, Africa and the Mediterranean area [1, 2]. Because of the trend of Mediterranean area towards global desertification and decline of water resources, *O*. *ficus-indica* has an interesting potential as fruit and vegetative crop. The various vegetative parts of *O*. *ficus-indica* have marked economic importance, and are exploited as foods and pharmaceuticals [3]. Cactus fruits (prickly pear) are used for manufacturing juices, jams, jellies and alcoholic beverages. Young cladodes (stem segments) are commercialized mainly as minimally processed fresh foods [4]. Although less valuable than fruit crops, the interest towards vegetative crop of *O*. *ficus*-*indica* increased in the last decades. Cladodes were exploited for nutraceutical and health-promoting properties [5]. Since ancient times, *O*. *ficus*-*indica* was used in traditional folk medicine, and, recently, the popularity of cladodes increased also in developed countries, being recognized as source of phytochemicals and prebiotics [6]. Due to the high annual productivity of biomass per hectare (10–40 tones dry weight), cladodes represent a cheap and suitable substrate for functional foods or dietary supplements [7]. Several studies showed the presence of natural compounds having anti-inflammatory, antioxidant, hypoglycemic, antimicrobial and neuro-protective activities [5, 8]. The therapeutic potential of cladode extracts was shown *in vitro* or *in vivo*. A number of diseases were treated, including metabolic syndromes (diabetes type 2 and obesity), rheumatism, cerebral ischemia, renal diseases, cancers, and viral and bacterial infections [5, 9–11]. Also the prebiotic activity of cladodes-derived oligosaccharides was shown [6, 11]. Cladodes are a source of phenolics, fibers, polyunsaturated fatty acids and vitamins [5, 8]. Cladode extracts were used for technological applications as dietary supplements, ingredients for beverages, breakfast cereals and margarine or thickening agents in vegetable soups and dessert gels [11, 12]. The cosmetic application is also wide. Cladode-gel exert a healing effect on dermal wounds [13].

The enhancement of the biogenic activity of cactus cladodes may rely into standardized and marketable products, having well-known or novel applications. For this purpose, bioprocessing of plant materials through microbial fermentation received considerable interest because of the better capacity to exploit inherent bioactivities with respect to industrial enzymatic processes. The only few studies investigated the effect of yeast fermentation on the health-promoting features of prickly pear cladodes [14]. To the best of our knowledge, no studies have considered the potential of the lactic acid fermentation. Besides, the identification and characterization of inherent components of *O*. *ficus-indica* were largely investigated [5, 8] but very limited information are available regarding the metabolites that are generated during fermentation.

This study aimed at investigating the capacity of selected lactic acid bacteria to enhance the antioxidant and immune-modulatory features of cactus cladodes with the perspective of producing novel functional foods, dietary supplements or pharmaceutical preparations.

## Material and Methods

### Microorganisms and culture conditions

Thirteen strains lactic acid bacteria, belonging to the Culture Collection of the Department of Soil, Plant and Food Science, University of Bari Aldo Moro, Bari, Italy, were used as starters for fermentation (Table 1). All strains were previously isolated from fruits and vegetables. Strains were identified by partial sequencing of the 16S rRNA, *recA*, *pheS*, and *rpoA* genes. Cultures were maintained as stocks in 15% (vol/vol) glycerol at -80°C and routinely propagated at 30°C for 24 h in MRS broth (Oxoid, Basingstoke, Hampshire, United Kingdom). Almost all the above strains were previously characterized for technology (e.g., acidifying capacity, growth and sensory profile) and functional features [15–23].

**Table 1:** Lactic acid bacteria strains (n = 13) used in this study.

Strain	Source	Reference
*Lactobacillus plantarum* CIL6	Cherry	[19]
*L*. *plantarum* POM1	Tomato	[16]
*L*. *plantarum* 1MR20	Pineapple	[18]
*Lactobacillus brevis* POM2	Tomato	[16]
*L*. *brevis* POM4	Tomato	[16]
*Lactobacillus fermentum* F1	Freanch beans	[15]
*Lactobacillus rossiae* 2LC8	Pineapple	[18]
*Lactobacillus curvatus* PE5	Pepper	[17]
*Pediococcus pentosaceus* F10	Freanch beans	[15]
*P*. *pentosaceus* CILSWE5	Cherry	[19]
*Leuconostoc mesenteroides* KI6	Kiwi fruit	Di Cagno (unpublished observations)
*Weissella cibaria confusa* POM12	Tomato	[16]
*W*. *cibaria/confusa* P9	Papaya	[20]

### Cladode pulp processing and fermentation

Fresh cladodes of *Opuntia ficus-indica* (L.) Mill. (genotype Sanguigna) were collected in the autumn 2014 from an organically certified farm (Azienda agricola Ferarro Biofarm, Cod. Op. SUOLOESALUTE ASS34739AG7102), which is located in Santa Maria di Belice (Sicily, Italy). The owner of the farm gave permission to carry out the analyses on fresh cladodes. This study did not involve endangered or protected species. During the edible stage with tender and crispy structure, young cladodes were harvested three montgs after sprouting, before developing spines. After harvesting, cladodes were washed with water, cut into strips and blended by vertical food processor (mod R8 Robot Coupe, Bologna, Italy). Resulting cladode pulp (CP) was thoroughly mixed to ensure representative samples and stored at −20°C until processing and analyses. Lactic acid bacteria strains were singly used as starters. Cells were cultivated in MRS broth until the late exponential growth phase was reached (ca. 12 h), washed twice in 50 mM phosphate buffer, pH 7.0, and re-suspended in the CP at the initial cell density of ca. Log 8 CFU/g. CP was fermented at 30°C for 24 h. Samples were taken before and after fermentation. CP, without bacterial inoculum and chemically acidified with lactic acid (final pH of ca. 4.0), was incubated under the same conditions and used as the control (CP-CT).

### Kinetics of growth and acidification

Growth was monitored by plating on MRS agar. The pH was measured by a Foodtrode electrode (Hamilton, Bonaduz, Switzerland). Kinetics of growth were determined and modeled according to the Gompertz equation as modified by Zwietering et al. [24]: *y* = *k* + *A* exp{-exp[(μ_max_ *e*/*A*)(λ - *t*) + 1]}, where *k* is the initial level of the dependent variable to be modeled (Log CFU/ml), *A* is the difference in cell density between inoculation and the stationary phase, μ_max_ is the maximum growth rate (expressed as Log CFU/ml/h), λ is the length of the lag phase (expressed in hours), and *t* is the time. Experimental data were modelled by the non-linear regression procedure of the Statistica 8.0 software (Statsoft, Tulsa, USA).

### Determination of sugars, organic acids and free amino acids

Equal volumes of perchloric acid (5%, vol/vol) were added to fermented and unfermented CP aliquots as precipitating agent. The suspension was kept at 4°C overnight, centrifuged at 10,000 × g, 10 min, and filtered through a Millex-HA 0.22-mm pore size filter (Millipore Co., Bedford, MA). The concentration of glucose, fructose and sucrose was determined through HPLC analysis, using an ÄKTA Purifier system (GE Healthcare) equipped with a Spherisorb column (Waters, Millford, USA) and a Perkin Elmer 200a refractive index detector. Elution was at 32°C with a flow rate of 1 ml/min, using acetonitrile 80% as the mobile phase [25]. Organic acids were determined by HPLC, using an ÄKTA Purifier system (GE Healthcare) equipped with an Aminex HPX-87H column (ion exclusion, Biorad) and a UV detector operating at 210 nm. Elution was at 60°C with a flow rate of 0.6 ml/min, using 10 mM H_2_SO_4_ as the mobile phase [26]. Peaks were identified by comparing elution times and spiking samples with known quantities of standard solutions of acetic and lactic acid. Total and individual free amino acids were analyzed by a Biochrom 30 series Amino Acid Analyzer (Biochrom Ltd., Cambridge Science Park, England), with a Na-cation-exchange column (20 by 0.46 cm inner diameter) as described by Rizzello et al. [27].

### DPPH radical scavenging activity

The antioxidant activity of fermented and unfermented CP was assayed as radical scavenging activity on 1,1-diphenyl-2- picrylhydrazyl radical (DPPH˙). Analyses were carried out using water-soluble (WSE), ethyl acetate-soluble extracts (ESE) or hexane-soluble extracts (HSE) from raw CP, CP-CT and fermented CP. For WSE preparation, 50 g of sample were freeze-dried, re-suspended in 50 ml of distilled water, and incubated at room temperature for 30 min under stirring conditions. Suspensions were centrifuged at 14,000 × g for 20 min to recover the supernatants for scavenging activity assay. ESE were obtained as described by Filannino et al. [23], with some modifications. Fifty grams of sample were freeze-dried and mixed with 120 ml of aqueous methanol (70%, vol/vol). The mixture was shaken for 1 h and centrifuged at 4,225 × g for 10 min. The supernatant was removed, and the residue was further extracted as described above. Methanol was evaporated under vacuum at 30°C, and solids were re-dissolved with 30 ml of Milli-Q water and acidified to pH 1.5 with hydrochloric acid. The liquid-liquid extraction was carried out with 120 ml of ethyl acetate. The mixture was shaken every 10 min for 30 min. The liquid-liquid extraction was repeated, and the extract was evaporated under vacuum at 30°C. Solids were re-dissolved in 50 ml of methanol. HSE were prepared using the method described by Kubola et al. [28]. Fifty grams of sample were freeze-dried, placed in a vessel protected from light, and mixed with 100 ml of extraction solvent (hexane/acetone/ethanol: 50:25:25 vol/vol/vol). The mixture was magnetically stirred for 30 min, and then 15 ml of water were added. The upper layer was placed in a round-bottomed flask, and evaporated to dryness. The residue was dissolved to a final volume of 50 ml. Free radical scavenging capacity was determined using the stable 2,2-diphenyl-1-picrylhydrazyl radical (DPPH˙), as reported by Yu et al. [29]. The reaction mixture was prepared by diluting WSE and ESE in methanol, and HSE in acetone. The reaction was monitored by reading the absorbance at 517 nm every 2 min for 30 min through a spectrophotometer. HSE were analyzed in a quartz cuvette. A blank reagent was used to verify the stability of DPPH˙ over the test time. The absorbance value measured after 10 min was used for the calculation of the scavenging activity by extracts. Scavenging activity was expressed as follows: DPPH scavenging activity (%) = [(blank absorbance–sample absorbance) / blank absorbance] × 100. Butylated hydroxytoluene (BHT) 75 ppm was used as the antioxidant reference. Reaction mixture was prepared by diluting BHT in methanol or acetone. WSE were also subjected to protein hydrolysis with trypsin (EC 3.4.21.4; Sigma-Aldrich Co.), as described by Atanassova et al. [30], and DPPH radical scavenging activity was also assayed on digested WSE as described above. Further analyses were carried out only for the best performing strains.

### Purification of antioxidant compounds

Peptides from WSE were analyzed through Reversed-Phase Fast Performance Liquid Chromatography (RP-FPLC), using a Resource RPC column and an ÄKTA FPLC equipment, with the UV detector operating at 214 nm (GE Healthcare Bio-Sciences AB, Uppsala, Sweden). Aliquots of WSE, containing ca. 1 mg/ml of peptides, were added to 0.05% (vol/vol) trifluoroacetic acid (TFA) and centrifuged at 10,000 × g for 10 min. The supernatant was filtered with a 0.22 μm pore size filter and loaded onto the column. Gradient elution was carried out at the flow rate of 1 ml/min, using a mobile phase composed of water and CH_3_CN, containing 0.05% TFA. The concentration of CH_3_CN was increased linearly from 5 to 46% between 16 and 62 min, and from 46 to 100% between 62 and 72 min. Fractions (2 ml) were recovered by using a FRAC 920 automatic fraction collector (GE Healthcare). Solvents were removed from collected fractions by freeze drying. The fractions were re-dissolved in distillated water and subjected to assays for antioxidant activity. The concentration of peptides in WSE and purified fractions was determined by the *o*-phthaldialdehyde (OPA) method [31]. Free phenolic acids and flavonoids in ESE were analyzed through High Performance Liquid Chromatography (HPLC) using an Ultimate 3000 system equipped with a column Discovery C18 (250mm×4.6mm; 5μm). Solvent A (water/formic acid, 99.5/0.1, vol/vol) and B (methanol/formic acid, 99.5/0.1, vol/vol) were used for chromatographic separation. Samples were eluted with the following gradient: starting with A:B; 85:15 vol/vol, then linear gradient to 70% B in 25 min, then linear gradient till 95% B in 35 min maintained at 95% B for 5 min and equilibrate to initial mobile phase in 5 min. Twenty microliters of ESE were injected, and elution was carried out at 35°C with a flow rate of 1 ml/min. A scan mode ranging from 245 to 550 nm wavelength was used. Phenolic acids and flavonoids were detected at 280 and 360 nm, respectively. Flavonol aglycons (kaemferol and isorhamnetin), were identified and quantified using pure standards purchased from Sigma-Aldrich (Steinheim, Germany) by comparison of retention time and UV absorbance spectrum. Fractions were recovered every two minute. Solvents were removed from collected fractions and dried through a Speed-Vac centrifuge (Thermo Scientific, Waltham, MA) at 30°C. The fractions were re-dissolved in methanol and subjected to assays for antioxidant activity. The concentration of total phenols in ESE and purified fractions was determined as described by Slinkard and Singleton [32]. Data were expressed as equivalent of gallic acid.

### Total carotenoids analysis

Total carotenoids content of CP was determined by measuring the absorption of HSE at 450 nm [28]. HSE were analyzed in a 1.5 mL quartz cuvette, through an UV–vis spectrophotometer. Total carotenoids concentration was calculated as equivalent of lutein.

### Vitamin C analysis

Determination of vitamin C was done according to [33]. Briefly, extraction from sample was done using metaphosphoric acid solution. A reducing solution was used to transform dehydro L(+) ascorbic acid into L (+) ascorbic acid. Total L (+) ascorbic acid content was determined by the HPLC system Ultimate 3000 (Dionex, Germering, Germany) equipped with photodiode array detector (PAD 3000), low pressure pump Ultimate 3000, injector loop Rheodyne (Rheodyne, USA, volume 20 μl), column Ascentis RP Amide (250 mm × 4.6 mm; 5 μm), and column oven. Chromeleon Software vs 6.8 (Dionex, Germering, Germany) was used to perform the analysis and to elaborate the data. Solvent A (50 mM H_3_PO_4_, pH 3) and B (methanol) were isocratic eluted in 10 min for chromatographic separation. Twenty microliters of extract were injected and elution was carried out at 25°C with a flow rate of 1 ml/min. The analyses of L (+) ascorbic acid (Sigma-Aldrich, Steinheim, Germany) were performed at UV wavelengths of 245 nm. A scan mode ranging 230–450 nm was used. Quantity of ascorbic acid was expressed as mg/100 g of fresh weight.

### Caco-2/Tc7 cell culture

Human intestinal Caco-2 cells (TC7 clone) [34] supplied by the Istituto Superiore di Sanità (Rome, Italy) were routinely cultured in Dulbecco Modified Eagle’s Medium supplemented with glucose (4.5 g/l), 2 mM L-glutamine, 1% (wt/vol) non-essential amino-acids, 50 μg/ml penicillin/streptomycin and 10% (wt/vol) thermally inactivated fetal calf serum. Cells were maintained in 25 cm^3^ culture flasks at 37°C under 95% of humidified atmosphere and 5% CO_2_. All the chemicals were supplied from Hyclone (Cramlington, UK). Cells were routinely passed every 7 days (Falcon, Free Lake, NJ) and the medium was changed at least twice a week. Passage was performed at 80% of confluence. Experiments were carried out on passages from 68 to 85. Caco-2/TC7 cells were seeded in 96-well plates at the density of 5 x 10^3^ cells per well. Further, cells were allowed to reach 70% of confluence in about 3 days and then treated for 24 h. Cell viability was measured using the vital dye Neutral Red (NR) uptake assay [35].

### Nitric oxide measurements

Nitric oxide (NO) was determined by measuring the stable oxidation products nitrite and nitrate [36]. Caco-2/TC7 cells were seeded in 24-well plates by re-suspending on DMEM medium (Gibco, BRL, Gaithersburg, MD, USA), without phenol red, at the density of 3.0 x 10^4^ cells per well and cultivated for 5 days at 37°C. After cultivation, different CP extracts (10 mg/ml) alone or in combination with a cytomix solution (TNF α, 100 ng/ml; IL-1β 5 ng/ml; INF γ, 200 U/ml) were added to DMEM and cells were further treated for 48 h. Cytomix solution was added to induce oxidative stress. When added alone, citomix was used as positive control. Diluted CP extracts were sterilized through 0.22 μm filter membrane (Millipore) to remove lactic acid bacteria cells. After 48 h of incubation, cell culture supernatants were first incubated for 1 h with nitrate reductase to convert nitrate to nitrite and then mixed with an equal volume of Griess reagent (Sigma) (1%, wt/vol, sulphanilic acid in 0.5 N HCl and 0.1%, wt/vol, N^1^-1-napthylethylendiamine hydrochloride) and the absorbance at 540 nm was measured after 60 min [36]. The nitrite concentration was determined by reference to a standard curve of sodium nitrite. The percentage inhibition was calculated based on the ability of WSE, ESE, or HSE from CP unstarterd, CP-CT and CP fermented with lactic acid bacteria to inhibit NO formation by cells compared with the control (cells in DMEM medium without CP extracts containing respective solvents water, ethyl-acetate or hexane), which was considered as 0% inhibition. All these treatments did not alter cell viability as shown by NRU assay.

### Measurement of transepithelial electrical resistance (TEER) of Caco-2/TC7 cells

To allow differentiation, Caco-2/TC7 cells were seeded (1 x 10^4^ cells/mL) onto 12-well insert plates with polyethylene terepthlate (PET) membrane (pore size of 0.4 μm) (BD Falcon Franklin Lakes, NJ USA) and cultivated for 21 days at 37°C. After cultivation, cells were treated for 48 h with CP extracts alone or in combination with IL-1β (25 ng/ml). DMEM containing methanol (0.5%, vol/vol) and ethanol (0.5%, vol/vol) were used as the negative controls. DMEM containing IL-1β (25 ng/ml) was used as the positive control. The integrity of monolayer was monitored by measuring the transepithelial electric resistance (TEER) through the Millicell-ERS device (Millipore, Bedford, MA, USA). Measurements were expressed in Ohms x cm^2^, after subtracting the mean values of the resistance from cell-free inserts. TEER data were recorded at room temperature.

### Permeability measurement

Caco-2 cells were seeded at a density of 300 x 10 ^3^ cells/cm^2^ on polycarbonate inserts (0.4 mm pore diameter, 0.9 cm^2^ area). After 21 days, differentiated cells were treated from the apical compartment with WSE, ESE, or HSE from different CP samples. Fluorescein isothiocyanate-conjugated dextran (FITC-dextran; MW, 4.4 kd; Sigma, St. Luis, MO) was dissolved in culture medium and used at a final concentration of 2.2 mg/ml in the apical cell compartment. After 3 h of incubation the amount of fluorescence was measured in the basal compartment with a spectrofluorometer. The excitation and emission wavelengths were 490 and 520 nm, respectively.

### Interleukin-8 (IL-8) and tumor necrosis factor alfa (TNFα) detection

Caco-2/TC7 cells were incubated for 24 h at 37°C with INF-γ (2 ng/mL) (Peprotech) and then stimulated for other 24 h with WSE, ESE, or HSE from CP samples at the concentration of 10 mg/ml. DMEM containing methanol (0.5%, vol/vol) and ethanol (0.5%, vol/vol) were used as the negative controls. Synthesis of the pro-inflammatory IL-8 and TNFα was measured using the enzyme-linked immunosorbent assay (ELISA) (Bender MedSystems, Vienna, Austria). Quantification was carried out using a reference standard curve as provided by manufacturer.

### Detection of intracellular reactive oxygen species (ROS)

The level of intracellular ROS was assessed by measuring the oxidation of the probe 2′,7′-dichlorofluorescin diacetate (DCFH-DA) (Molecular Probes, Lifesciences), according to the method of Cathcart et al. [37]. Caco-2/TC7 cells were seeded in a 96-well microplate (5 × 10^3^ cell/well) in DMEM high glucose medium and allowed to reach 80% of confluence. Cells were treated with WSE, ESE or HSE from raw CP, CP-CT and CP fermented with lactic acid bacteria diluted in culture medium and co-incubated for additional 24 h with IL-1β (25 ng/ml). DMEM high glucose medium was used as the control. After incubation, CP extracts were removed and cells were washed twice with phosphate buffered saline (PBS) solution. Further, medium was replaced in the dark environment with DCFH-DA (25 μM in PBS), then cells were incubated for 30 min. After incubation, the cells were washed and fluorescence was measured with a FL800 microplate fluorescent reader (Bioteck Instruments, USA) at excitation/emission wavelengths of 485/528 nm. Values were expressed as fluorescence intensity (Fi) units.

### Prostaglandin E2 (PGE2) quantification

PGE2 concentration was determined in the culture medium of differentiated Caco-2 cell monolayers after 24 h of incubation by using the PGE2-monoclonal enzyme immunoassay kit, as recommended by the manufacturer (Cayman Chemicals).

### Statistical Analysis

Data were subjected to one-way ANOVA; pair-comparison of treatment means was achieved by Tukey’s procedure at P0.05, using the statistical software, Statistica for Windows (Statistica7.0 per Windows). Data for vitamin C, total carotenoids, GABA, kaemferol, and isorhamnetin, antioxidant activity, nitric oxide release, TNFα, ROS, TEER, PGE2, IL-8 levels were subjected to permutation analysis using PermutMatrix.

## Results

### Cactus cladodes fermentation

Preliminarily, 13 strains of lactic acid bacteria, which were previously isolatedand identified from vegetables and fruits [15–20], and selected based on technology and functional features (Table 1), were assayed during single fermentation (30°C for 24 h) of cactus young cladode pulp (CP). After 24 h, *Lactobacillus fermentum* F1 and *Leuconostoc mesenteroides* K16 were the only strains that did not grow on CP. *Lactobacillus curvatus* PE5, *Pediococcus pentosaceus* F10, and *Weissella cibaria* POM12 and P9 showed an increase of the cell number by only ca. one half log cycle (from ca. 8.0 ± 0.3 to 8.3 ± 0.05–8.7 ± 0.03 Log CFU/ml). On the contrary, *Lactobacillus plantarum* CIL6, POM1 and 1MR20, *Lactobacillus brevis* POM2 and POM4, *Lactobacillus rossiae* 2LC8 and *P*. *pentosaceus* CILSWE5 grew from ca. 8.0 to 9.24 ± 0.2–9.58 ± 0.1 Log CFU/ml. Overall, the lag phase ranged from 5.96 ± 0.24 to 9.81 ± 0.28 h, and μ_max_ varied from 0.18 ± 0.012 to 0.25 ± 0.019 Log CFU/ml/ h. CP had an initial value of pH of 4.3 ± 0.18. After fermentation, the value of pH remained almost constant or decreased to ca. 4.05. *L*. *plantarum* CIL6, POM1 and 1MR20 were the only strains, which decreased the value of pH to ca. 3.98. After fermentation, CP, without bacterial inoculum, contained ca. 2.5 and 3.0 Log CFU/ml of presumptive lactic acid bacteria and yeasts, respectively. Fermented CP contained ca. 1.0 Log CFU/ml of yeasts.

Based on the above data, further analyses on antioxidant activity, total phenols, vitamin C and total carotenoids were carried out only on CP fermented with the best growing strains. Therefore, *L*. *fermentum* F1, *Leuc*. *mesenteroides* K16, *L*. *curvatus* PE5, *P*. *pentosaceus* F10, and *W*. *cibaria* POM12 and P9 were excluded. Raw CP had concentrations of glucose, fructose and malic acid of 8 ± 1, 26 ± 2, and 28 ± 1 mmol/kg, respectively. During fermentation, such compounds were almost depleted. The synthesis of lactic acid ranged from 86 to 92 mmol/kg. The only exceptions were *L*. *brevis* POM2 and POM4, and *L*. *rossiae* 2LC8, which synthesized 57 ± 1–64 ± 2 mmol/kg of lactic acid. Ethanol was found mainly during CP fermentation with *L*. *brevis* POM2 and POM4 (ca. 20 mmol/kg) and *L*. *rossiae* 2LC8 (18 ± 2 mmol/kg). Only traces of ethanol were found in CP fermented with *L*. *plantarum* POM1 and CIL6, and *P*. *pentosaceus* CILSWE5. Acetic acid was only found in traces.

After fermentation, total concentration of free amino acids (FAA) was the highest for CP fermented with *P*. *pentosaceus* CILSWE5, *L*. *rossiae* 2LC8 and *L*. *brevis* POM2 (597 ± 29–535 ± 29 mg/kg). CP fermented with *L*. *plantarum* CIL6, POM1and 1MR20 had the lowest concentration (302 ± 32–328 ± 29 mg/kg). Raw CP had a concentration of 467 ± 31 mg/kg and the control, without bacterial inoculum and chemically acidified (CP-CT), had a value of 453 ± 33 mg/kg. CP fermented with *L*. *brevis* POM2 and POM4 showed concentrations of glutamic acid (21.8 ± 5.8 and 21.0 ± 6.0 mg/kg, respectively) lower than those found in the other CP (51.5 ± 5.9–166.9 ± 7.2 mg/kg). γ-Amino butyric acid (GABA) was found in all fermented CP. The concentration ranged from 10.2 ± 0.8 to 11.9 ± 0.9 mg/kg. The only exceptions were found for *L*. *brevis* POM2 and POM4, which showed the highest level of GABA (45.7 ± 0.9 and 38.9 ± 0.9 mg/kg, respectively).

### Antioxidant activity and total phenols, vitamin C and total carotenoids

**Fig 1:**
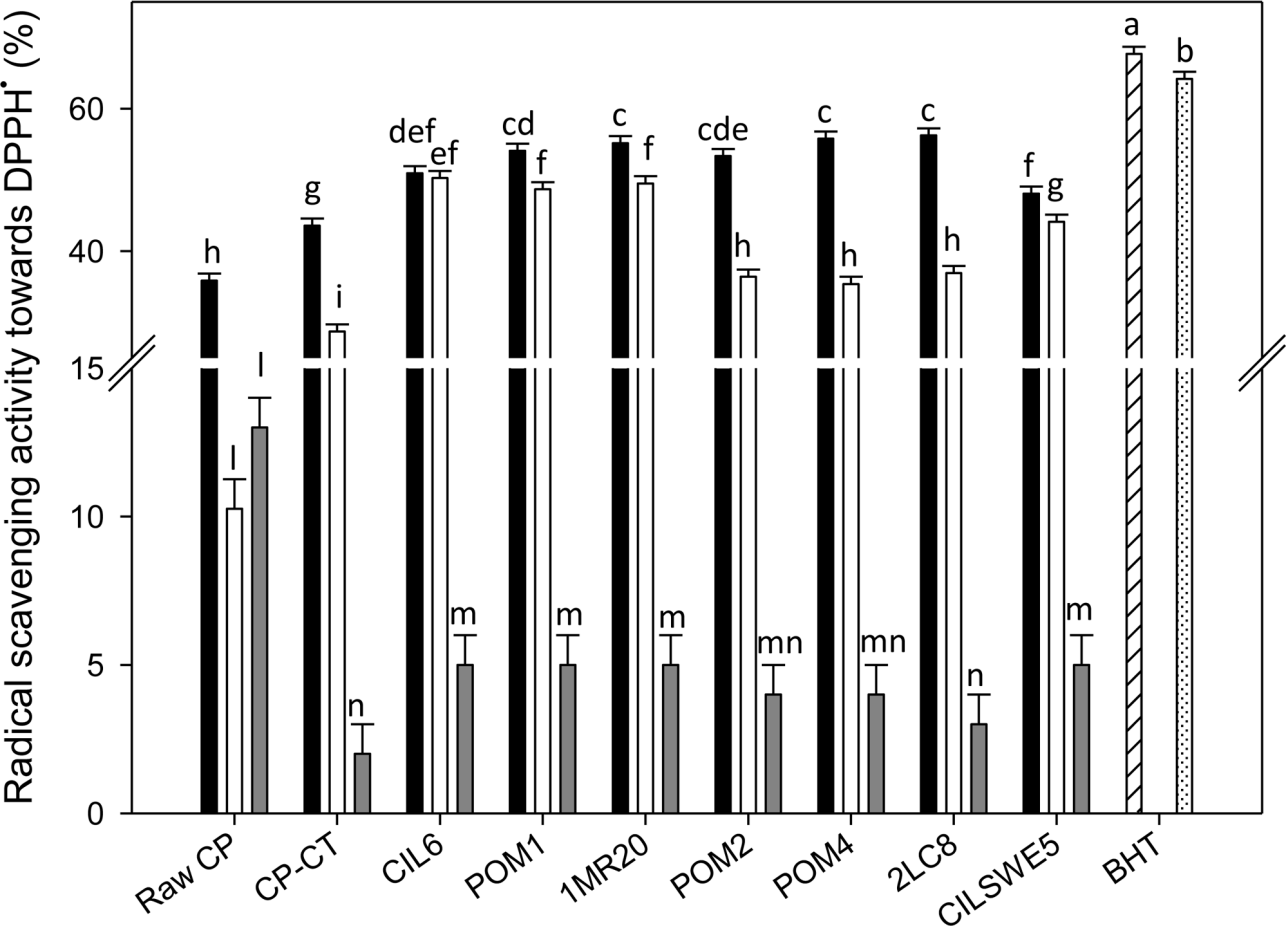
DPPH radical scavenging activity. DPPH radical scavenging activity of the water-soluble (black bars) (WSE), ethyl acetate-soluble (white bars) (ESE), and hexane-soluble (grey bars) (HSE) extracts from raw cladode pulp (raw CP), CP without bacterial inoculum and chemically acidified (CP-CT), and cladode pulp fermented with *Lactobacillus plantarum* POM1 (POM1), CIL6 (CIL6) and 1MR20 (1MR20), *Lactobacillus brevis* POM2 (POM2) and POM4 (POM4), *Lactobacillus rossiae* 2LC8 (2LC8) and *Pediococcus pentosaceus* CILSWE5 (CILSWE5). Butylatedhydroxytoluene (BHT) was used as positive control. The reaction mixture was prepared by diluting BHT in methanol (hatched bar) or acetone (dotted bar). Data are the means (± SD) of three independent experiments performed in triplicate. Data were subjected to one-way ANOVA; pair-comparison of treatment means was achieved by Tukey’s procedure at P0.05. Bars with different superscript letters differ significantly (P<0.05).

Antioxidant activity was assayed as radical scavenging activity on DPPH radical. Apart from the sample (raw CP, CP-CT and fermented CP), the type of solvent markedly affected the extraction of bioactive compounds. Therefore, the analysis was carried out using water-soluble (WSE), ethyl acetate-soluble (ESE) and hexane-soluble (HSE) extracts. During radical scavenging assay, the colored stable DPPH radical is reduced to non-radical DPPH-H, when in the presence of an antioxidant or a hydrogen donor. DPPH radical, without antioxidants, was stable over the time. Under the assay conditions, the 100% of activity corresponded to the complete scavenging of DPPH radical (50 μM final concentration) after 10 min of incubation with the antioxidant compounds. According to previous studies [38, 39], the color intensity of DPPH^•^ showed a logarithmic decline when it was in the presence of BHT. Apart from the extract used, the activity of raw CP and CP-CT was significantly (P<0.05) lower than that of BHT, which was used as the positive control. The radical scavenging activity of WSE, ESE and HSE from raw CP was 35.8 ± 0.56, 10.3 ± 0.41 and 13.5 ± 0.42%, respectively. WSE, ESE and HSE from CP-CT had a radical scavenging activity towards the stable radical DPPH of 43.6 ± 0.4, 28.7 ± 0.5 and 2.3 ± 0.1%, respectively (Fig 1).

Regardless of the strain used, the highest antioxidant activity was found for WSE, followed by ESE and HSE. Fermentation significantly (P<0.05) increased the radical scavenging activity of all WSE. Except for CP fermented with *P*. *pentosaceus* CILSWE5 (48.1 ± 0.41%) and *L*. *plantarum* CIL6 (50.9 ± 0.38%), all the other strains caused a remarkable increase of the radical scavenging activity (53.4 ± 0.24–56.2 ± 0.51%). Compared to raw CP (10.3 ± 0.41%) and CP-CT (28.7 ± 0.53%), also the ESE from fermented CP showed a higher increase of the antioxidant activity. The highest values were found for *L*. *plantarum* CIL6, POM1 and 1MR20 (48.7 ± 0.12, 49.5 ± 0. 22 and 50.3 ± 0.31%, respectively). Although the antioxidant activity from HSE was the lowest, the positive effect of the fermentation was confirmed also in this case (Fig 1).

The analysis of total phenols, water soluble vitamin C and total carotenoids was carried out using ESE, WSE and HSE, respectively [28, 32, 33].

Total phenols of raw CP accounted for 37.4 ± 1.2 mg gallic acid equ/100 g of fresh weight. The concentration of total phenols from CP fermented with *L*. *plantarum* CIL6, POM1 and 1MR20 and *P*. *pentosaceus* CILSWE5 did not significantly (P>0.05) vary compared to raw CP. After fermentation with *L*. *brevis* POM2 and POM4, and *L*. *rossiae* 2LC8, a slight decrease was found. The concentrations of vitamin C and total carotenoids from fermented CP agreed with antioxidant activity (Table 2). The concentration of vitamin C of raw CP was 9.8 ± 0.8 mg/100 g fresh weight. Chemical acidification decreased the concentration of vitamin C of CP (3.1 ± 0.2 mg/100 g), whereas lactic acid fermentation had a preserving effect. The vitamin C concentration of fermented CP ranged from 5.75 ± 0.2 to 8.4 ± 0.3 mg/100 g. The trend was similar for total carotenoids.

**Table 2:** Vitamin C and total carotenoids.

Sample	Vitamin C (mg/100 g fresh weight)	Total carotenoids (μg lutein equ/100 g fresh weight)
Raw CP	9.8 ± 0.9^a^	98.2 ± 1.9^a^
CP-CT	3.2 ± 0.2^e^	36.1 ± 1.8^e^
*Lactobacillus plantarum* CIL6	8.4 ± 0.3^a^	59.3 ± 1.5^c^
*L*. *plantarum* 1MR20	7.2 ± 0.1^b^	63.2 ± 1.2^c^
*Lactobacillus brevis* POM4	6.9 ± 0.1^c^	61.8 ± 0.9^c^
*Lactobacillus rossiae* 2LC8	5.8 ± 0.2^d^	46.4 ± 1.4^d^
*Pediococcus pentosaceus* CILSWE5	7.3 ± 0.2^b^	69.5 ± 0.9^b^

Notes: Vitamin C (mg/100 g of fresh sample) and total carotenoids (μg lutein equ/100 g of fresh weight) in raw cladode pulp (raw CP), chemically acidified cladode pulp (CP-CT), and cladode pulp fermented with selected lactic acid bacteria strains. Data are the means (± SD) of three independent experiments performed in triplicate. Data were subjected to one-way ANOVA; pair-comparison of treatment means was achieved by Tukey’s procedure at P0.05. Means within the column with different superscript letters differ significantly (P<0.05).

### Purification and identification of antioxidant compounds

**Fig 2:**
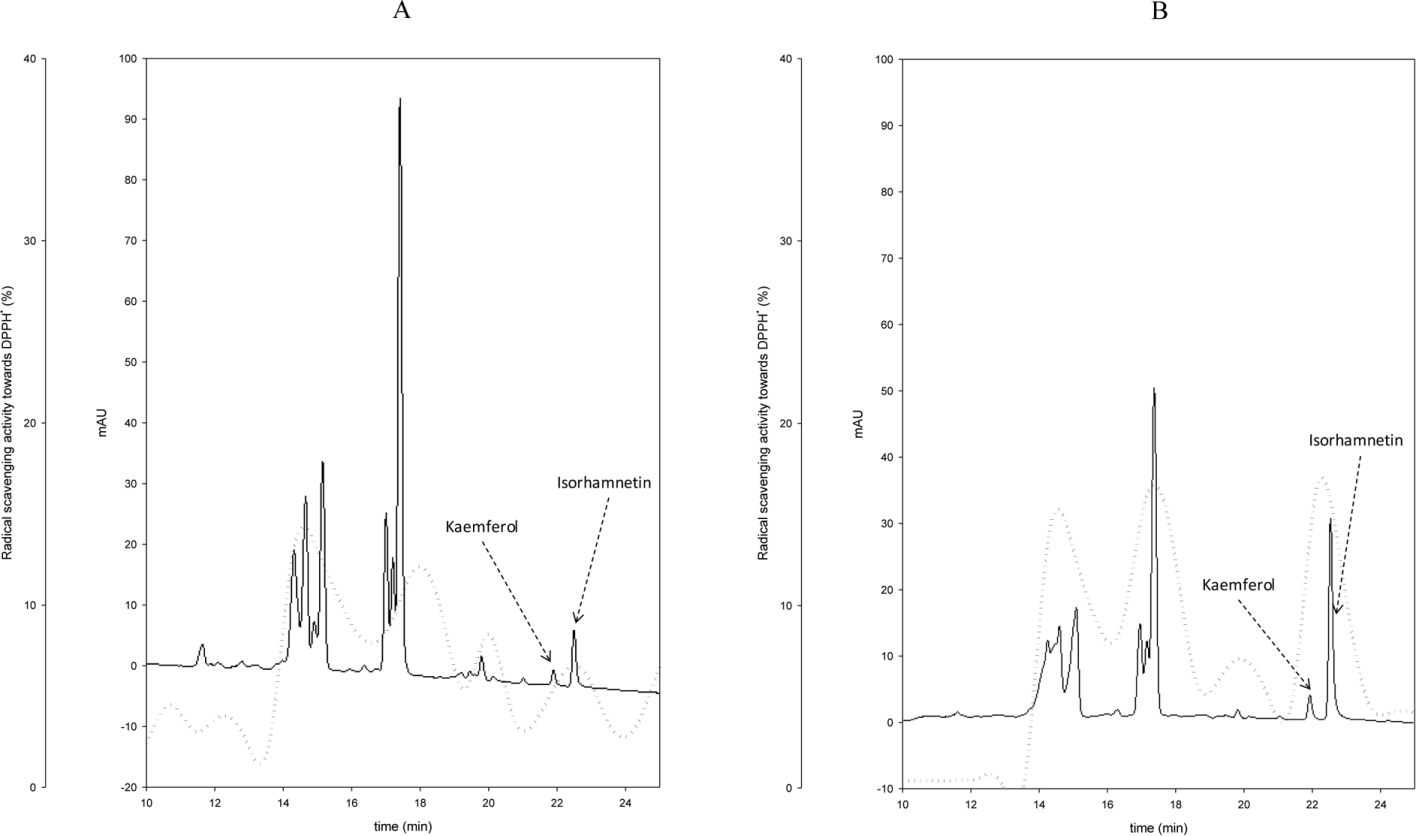
Flavonoid profiles of ESE. High Performance Liquid Chromatography (HPLC) profiles of ethyl acetate-soluble extracts (ESE) from cladode pulp (CP) without bacterial inoculum and chemically acidified control (CP-CT) (A) and CP fermented with *Lactobacillus plantarum* 1MR20 (B). The dashed line refers to the percentages of DPPH radical scavenging activity.

To further investigate the radical scavenging activity of CP, WSE from fermented CP were digested with trypsin. After digestion, the antioxidant activity decreased between ca. 6 to 32% depending on the strain (S1 Fig). These findings suggested that protein compounds were somewhat related to activity. Aiming at purifying the antioxidant compounds, WSE was subjected to fractionation through RP-FPLC, and resulting fractions were assayed for antioxidant activity. Since the antioxidant activity of WSE was distributed within a large number of fractions (data not shown), no further peptide identification was carried out. Based on these results, it was hypothesized a non-specific effect of CP protein derivatives, which were liberated through proteolysis during lactic acid bacteria fermentation. Aiming at identifying the antioxidant compounds from ESE, extracts were subjected to fractionation through HPLC analysis. The phenolic acid profiles of ESE from CP-CT and fermented CP were almost similar (data not shown). On the contrary, the flavonoid profiles of ESE from all fermented CP were diverse (Fig 2). Compared to CP-CT, the areas of two main peaks increased, which corresponded to kaemferol (retention time of 21.9 min) and isorhamnetin (retention time of 22.5). The concentration of kaemferol and isorhamnetin of CP-CT was 0.13 ± 0.04 and 0.55 ± 0.09 μg/g, respectively. The increases were strain dependent. The highest concentrations were found for CP fermented with *L*. *plantarum* 1MR20 (0.23 ± 0.05 and 2.1 ± 0.15 μg/g, respectively) and *L*. *brevis* POM4 (0.22 ± 0.03 and 2.0 ± 0.18 μg/g, respectively). For all the other strains the concentration of isorhamnetin ranged from 1.19 ± 0.07 to 1.36 ± 0.05 μg/g, whereas that of kaemferol did not significantly (P>0.05) vary compared to CP-Ct. Resulting fractions were also assayed for antioxidant activity. Compared to CP-CT, the highest increase of antioxidant activity was found in fractions matching with the retention times of kaemferol and isorhamnetin (Fig 2).

### Viability of Caco-2/TC7 cells

Preliminarily, the cytotoxicity of CP samples was checked using the Neutral Red (NR) uptake assay. Apart from the lactic acid bacterium used for fermentation, CP behaved similarly to DMEM (negative controls) and did not significantly (P>0.05) affect the Caco-2/TC7 cell proliferation up to 10 mg/ml.

### Nitric oxide (NO) release

**Fig 3:**
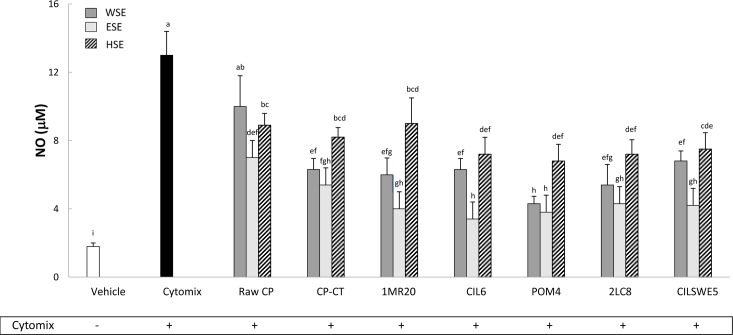
Nitric oxide (NO) (μM) release by Caco-2/TC7 cells. Caco-2/TC7 cells were treated for 48 h with water-soluble (WSE), ethyl-acetate (ESE), or hexane-soluble (HSE) extracts (10 mg/ml) from raw cladode pulp (raw CP), CP without bacterial inoculum and chemically acidified (CP-CT) and CP fermented with *Lactobacillus plantarum* 1MR20 (1MR20) and CIL6 (CIL6), *Lactobacillus brevis* POM4 (POM4), *Lactobacillus rossiae* 2LC8 (2LC8) and *Pediococcus pentosaceus* CILSWE5 (CILSWE5). Furthermore, cells were stimulated with a cytomix solution (TNFα, 100 ng/ml; IL-1β, 5 ng/ml; and INF γ, 200 U/ml). Data are the means (± SD) of three independent experiments performed in triplicate. Data were subjected to one-way ANOVA; pair-comparison of treatment means was achieved by Tukey’s procedure at P0.05. Bars with different superscript letters differ significantly (P<0.05).

Caco-2/TC7 cells were stimulated (48 h) with a cytomix solution containing TNF α, IL-1β and IFN-γ. Compared to the negative control (DMEM), the treatment significantly (P<0.05) increased the release of NO (Fig 3). On the contrary, treatments with WSE, HSE and, especially, ESE from raw CP, CP-CT and fermented CP markedly (P<0.05) inhibited the release of NO. Apart from the extract, the highest anti-inflammatory activity was found for CP fermented with *L*. *plantarum* CIL6 and *L*. *brevis* POM4.

### Transepithelial electric resistance (TEER)

**Fig 4:**
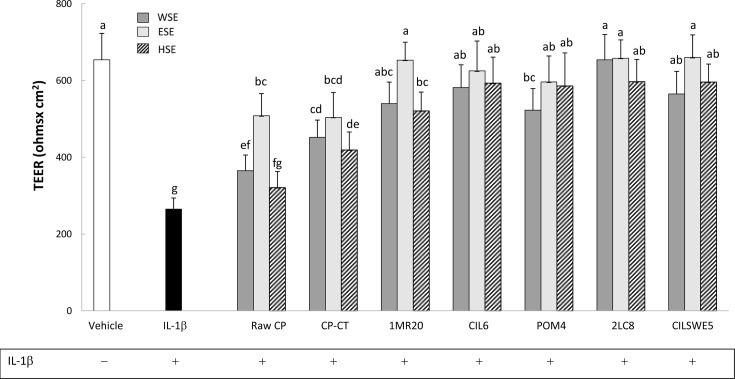
Transepithelial electric resistance (TEER) (Ohms x cm^2^) of Caco-2/TC7 cells. Caco-2/TC7 cells were incubated 24 h with IL-1β (25 ng/ml) and water-soluble (WSE), ethyl-acetate (ESE), and hexane-soluble (HSE) extracts (10 mg/ml) from raw cladode pulp (raw CP), CP without bacterial inoculum and chemically acidified (CP-CT) and CP fermented with *Lactobacillus plantarum* 1MR20 (1MR20) and CIL6 (CIL6), *Lactobacillus brevis* POM4 (POM4), *Lactobacillus rossiae* 2LC8 (2LC8) and *Pediococcus pentosaceus* CILSWE5 (CILSWE5). Data are the means (± SD) of three independent experiments performed in triplicate. Data were subjected to one-way ANOVA; pair-comparison of treatment means was achieved by Tukey’s procedure at P0.05. Bars with different superscript letters differ significantly (P<0.05).

Under culture conditions, Caco-2/TC7 cells develop morphological and functional characteristics of enterocytes, including intercellular tight junctions. Transepithelial electric resistance measured the integrity of the tight junctions. Preliminarily, TEER was measured in the presence of WSE, HSE or ESE from raw CP, CP-CT and fermented CP. During 48 h of incubation, TEER was not affected (data not shown). Treatment of Caco-2/TC7 cells with IL-1β (25 ng/mL) markedly (P<0.05) decreased the value of TEER (Fig 4). When Caco-2/TC7 cells were stimulated (basolateral compartment) with IL-1β and subsequently treated (apical compartment) with WSE, HSE or ESE from CP, the negative effect of IL-1β was markedly attenuated (P<0.05). Apart from the extract, all fermented CP showed the highest effect.

### Interleukin-8 (IL-8) and tumour necrosis factor alfa (TNFα) release

**Fig 5:**
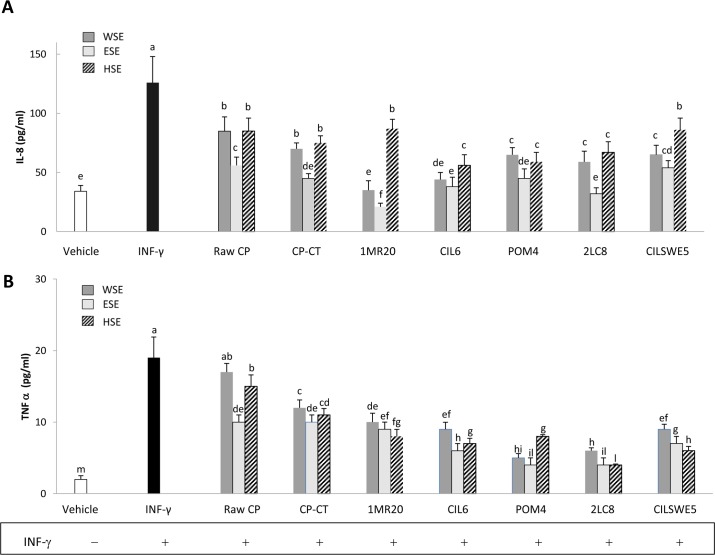
**Interleukin-8 (IL-8) (A) and tumour necrosis alfa (TNFα) (B) release by Caco-2/TC7 cells.** Caco-2/TC7 cells were stimulated for 24 h with interferon-gamma (INF-γ) (2 ng/mL) and subsequently treated with water-soluble (WSE), ethyl-acetate (ESE), or hexane-soluble (HSE) extracts (10 mg/ml) from raw cladode pulp (raw CP), CP without bacterial inoculum and chemically acidified (CP-CT) and CP fermented with *Lactobacillus plantarum* 1MR20 (1MR20) and CIL6 (CIL6), *Lactobacillus brevis* POM4 (POM4), *Lactobacillus rossiae* 2LC8 (2LC8) and *Pediococcus pentosaceus* CILSWE5 (CILSWE5). Data are the means (± SD) of three independent experiments performed in triplicate. Data were subjected to one-way ANOVA; pair-comparison of treatment means was achieved by Tukey’s procedure at P0.05. Bars with different superscript letters differ significantly (P<0.05).

IL-8 is a member of the C-X-C chemokine family and plays an essential role in the recruitment and activation of neutrophils, thereby initiating the inflammatory response. When Caco-2/TC7 cells were treated with INF-γ (2 ng/mL), a significant (P<0.05) increase of the synthesis of IL-8 and TNFα was found (Fig 5A and 5B). When Caco-2/TC7 cells were stimulated with INF-γ and also treated with WSE, HSE or ESE from raw CP, CP-CT and fermented CP, a significant (P<0.05) decrease of the synthesis of IL-8 and TNFα was found. Apart from the strain used, the synthesis of IL-8 and TNFα was more markedly inhibited (P<0.05) by treatment with ESE. The highest (P<0.05) inhibition of IL-8 was found with ESE from CP fermented with *L*. *plantarum* 1MR20 and *L*. *rossiae* 2LC8, whereas that of TNFα by ESE from CP fermented with *L*. *rossiae* 2LC8 and *L*. *brevis* POM4.

### Reactive oxygen species (ROS) scavenging

**Fig 6:**
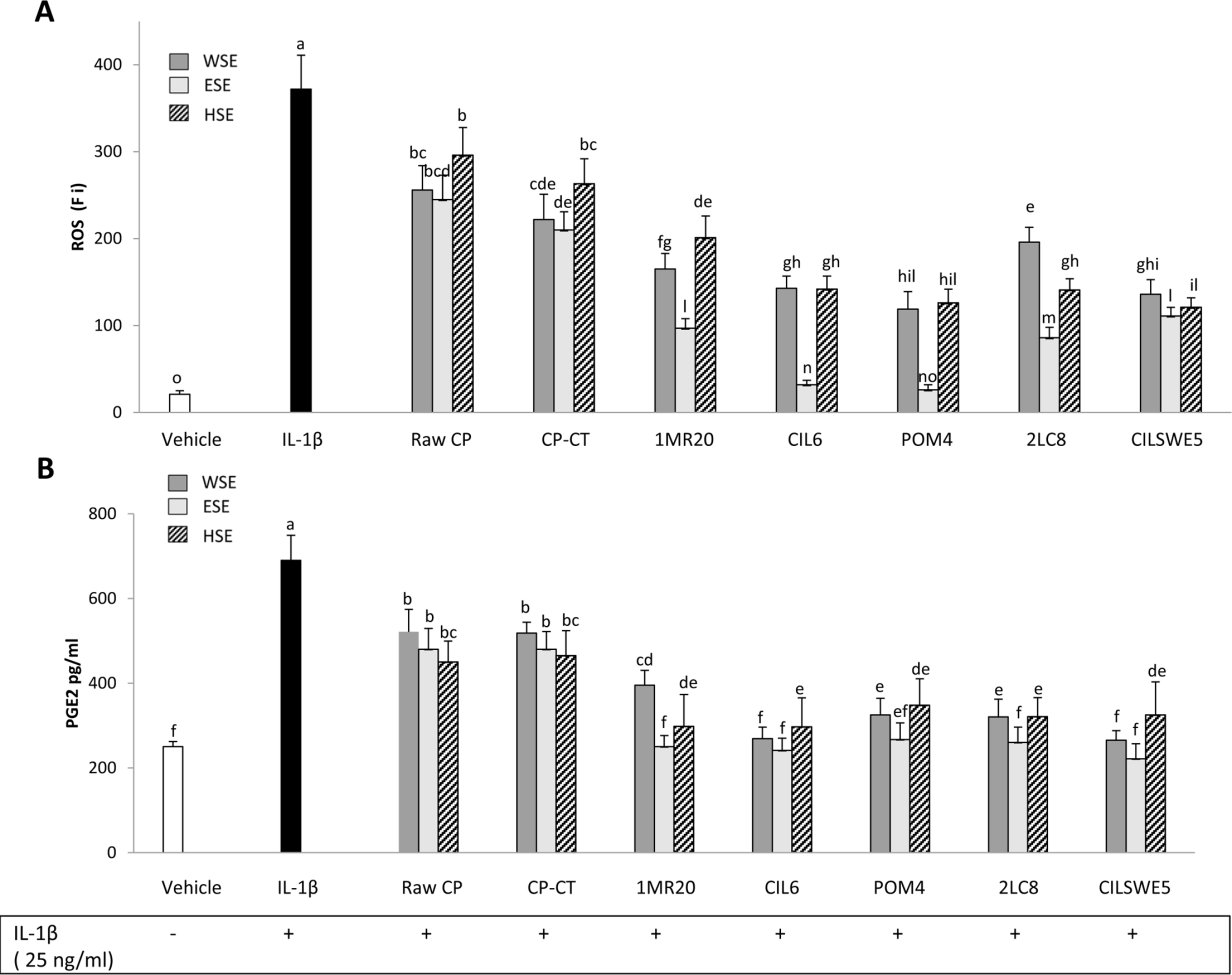
**Intracellular reactive oxygen species (ROS) (fluorescence intensity units, Fi) (A) and Prostaglandin E2 (PGE2) (pg/ml) (B) on Caco-2/TC7 cells.** Caco-2/TC7 cells were stimulated for 24 h at 37°C with IL-1β (25 ng/mL) and then treated for other 24 h with water-soluble (WSE), ethyl-acetate (ESE), or hexane-soluble (HSE) extracts (10 mg/ml) from raw cladode pulp (raw CP), CP without bacterial inoculum and chemically acidified (CP-CT) and CP fermented with *Lactobacillus plantarum* 1MR20 (1MR20) and CIL6 (CIL6), *Lactobacillus brevis* POM4 (POM4), *Lactobacillus rossiae* 2LC8 (2LC8) and *Pediococcus pentosaceus* CILSWE5 (CILSWE5). Only the best performing strains and representative species were considered. Data are the means (± SD) of three independent experiments performed in triplicate. Data were subjected to one-way ANOVA; pair-comparison of treatment means was achieved by Tukey’s procedure at P0.05. Bars with different superscript letters differ significantly (P<0.05).

The level of intracellular ROS was assessed by measuring the oxidation of the probe 2′,7′-dichlorofluorescin diacetate (DCFH-DA). When Caco-2/TC7 cells were subjected to treatment with IL-1β (25 ng/mL), a significant (P<0.05) increase of the intracellular level of ROS was found (Fig 6A). When Caco-2/TC7 cells stimulated with IL-1β were also treated with WSE, HSE or ESE from raw CP, CP-CT and fermented CP, a significant (P<0.05) decrease of the intracellular level of ROS was found. Apart from the strain used, the highest decrease was found by treatment with ESE. CP fermented with *L*. *plantarum* CIL6 and *L*. *brevis* POM4 had the highest effect.

The formation of inflammatory eicosanoids such as prostaglandin E2 (PGE2), which is synthesized from arachidonic acid by COX- 2, was further determined. Treatment of cells with IL-1β elicited markedly the synthesis of PGE2, whereas incubation with WSE, HSE or ESE from raw CP, CP-CT and, especially, fermented CP displayed high ability to decrease PGE2 accumulation in response to IL-1β (Fig 6B).

## Discussion

**Fig 7:**
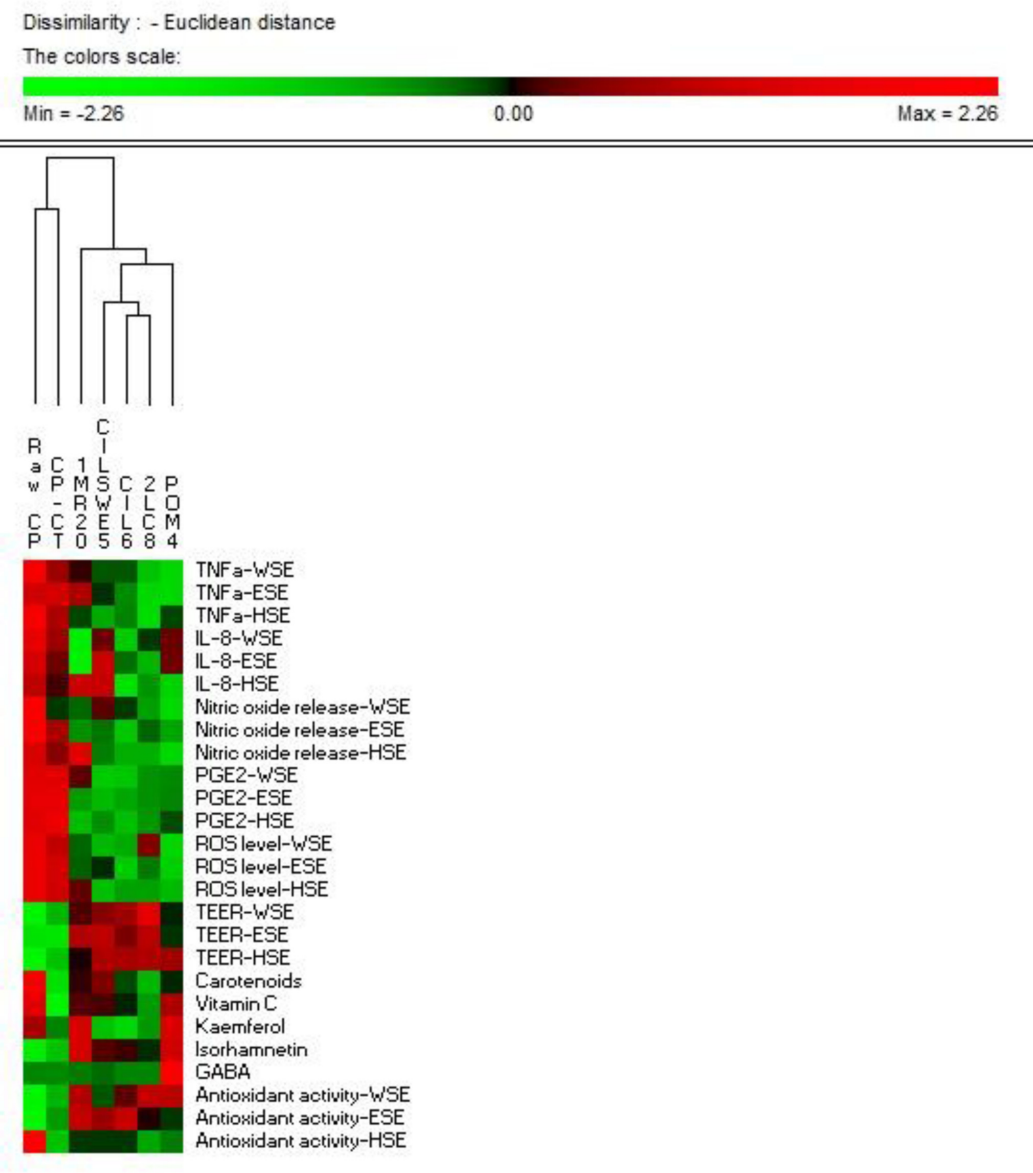
Permutation analysis of compositional and functional profiles. Permutation analysis of compositional [vitamin C, total carotenoids, γ-amino butyric acid (GABA), kaemferol, and isorhamnetin], and functional [antioxidant activity, nitric oxide release, tumour necrosis alfa (TNFα), reactive oxygen species (ROS), transepithelial electric resistance (TEER), Prostaglandin E2 (PGE2), and interleukin-8 (IL-8)] profiles of raw cladode pulp (CP), CP without bacterial inoculum and chemically acidified (CP-CT), and fermented CP with *Lactobacillus plantarum* 1MR20 (1MR20) and CIL6 (CIL6), *Lactobacillus brevis* POM4 (POM4), *Lactobacillus rossiae* 2LC8 (2LC8) and *Pediococcus pentosaceus* CILSWE5 (CILSWE5). Differences are represented colorimetrically with red and green indicating the highest and lowest values of the standardized data, respectively, for each parameter. All data were shown as a percentage of dissimilarity using Euclidean distance.

The role of lactic acid fermentation for improving the nutritional and functional features of many vegetables is well-known [40]. The health benefits mostly result via ingestion of microbial metabolites that are synthesized during fermentation. Lactic acid bacteria, intended as microbial cell factories, increase the functionality of many fermented vegetables through their enzyme portfolio that promotes the synthesis of various metabolites and/or the release of biogenic compounds, which are mainly cryptic in the raw matrix [41]. The high concentration of functional compounds and the intrinsic features of *Opuntia ficus-indica* L. fruits and, especially, cladodes may be suitable for preparations with health-promoting features [5, 8, 11]. First, this study investigated the potential of the lactic acid fermentation of cladodes. The functional features of fermented cladode pulp (CP) were compared to those of the raw CP (Fig 7). Thirteen strains of lactic acid bacteria, previously isolated from vegetable matrices that are particularly rich of polyphenols [15–20], were used as starters. The concentration of fermentable carbohydrates of CP was enough to allow the bacterial growth of seven out the thirteen strains. Usually, plant autochthonous lactic acid bacteria better adapt to plant environments compared to allochthonous strains coming from other sources[40]. The functional composition *O*. *ficus indica* varies depending on the plant organ [8]. Cladodes are good sources of amino acids and proteins [8]. Fermentation of CP with *L*. *brevis* POM2 and POM4 allowed the highest concentration of γ-amino butyric acid (GABA) (39–46 mg/kg), a non-protein amino acid with physiological functions (e.g., induction of hypotension, diuretic and tranquilizer effect) [42, 43]. These concentrations are above the physiological threshold (ca. 10 mg/day), thus hypothesizing an *in vivo* health benefits [44]. Usually, the capacity to synthesize GABA is strain specific. It confers resistance to bacterial cells under acidic conditions like those of CP [45]. Lactic acid fermentation exerted also a preservative effect on the levels of vitamin C and carotenoids, which are inherent in CP. Ascorbic acid is one of the most sensitive vitamins in foods. The stability of vitamin C varies depending on environmental factors such as pH, concentration of metal ions and redox state [46]. The decrease of pH is one of the main mechanisms to prevent ascorbate autoxidation when the redox potential changes [47]. Nevertheless, the slight decreases of pH that were found after fermentation suggested the involvement of other mechanisms. Phytochemicals such as ascorbic acid, carotenoids and phenols determined the marked radical scavenging activity of crude cladode cactus extracts [48]. The antioxidant activity of fermented cladodes was compared to that of a non-inoculated and chemically acidified control (CP-CT). Chemical acidification was done to exclude the effect of pH on the antioxidant activity and phenol extractability. As usual in herbal medicine, the solvent extraction of bioactive compounds from permeable solid plant materials is a key step to have phytochemical-rich products [8]. Water-soluble (WSE), ethyl acetate-soluble (ESE) and hexane-soluble (HSE) extracts were compared. The radical scavenging activity of CP was positively affected by lactic acid bacteria fermentation. The highest increase was found with ESE, suggesting a major role of ethyl acetate extractable compounds like phenols. When *L*. *plantarum* CIL6, POM1 and 1MR20 were used as starters, the DPPH radical scavenging activity was at least five and two times higher than that of raw CP and CP-CT, respectively.

Aiming at explaining the increase of antioxidant activity due to lactic acid fermentation, the contribution of various bioactive compounds was investigated. The preservative effect on vitamin C and carotenoids levels certainly gave a positive contribution to the activity from WSE and HSE. A consistent part of the antioxidant activity was lost after treatment with proteolytic enzyme, therefore, a further contribution by water-soluble peptides was not excluded. Nevertheless, two flavonoid aglycone derivatives, kaemferol and isorhamnetin, which were identified from ESE were clearly responsible for the increased radical scavenging activity. Flavonols, the most ubiquitous flavonoids in vegetables, are usually present at relatively low concentrations and under glycosylated forms [49]. Esterase activities towards glycosylated forms increases the concentration of flavonols, which accumulate in the outer and aerial tissues of Cactus (skin and leaves) because the light stimulation [8, 49]. Flavonol aglycones contain multiple hydroxyl free groups and possess higher antioxidant activity than their glycosides [49]. Furthermore, native glycosylated forms cannot be absorbed by the human organism, needing previous hydrolysis by intestinal enzymes or colonic microbiota. Aglycones are directly absorbed at the level of the small intestine [49]. Esterase enzymes are widespread in *L*. *plantarum* strains, allowing the metabolism of flavonol glycosides in plant matrices [50]. Several flavonoid derivatives such as kaempherol, quercetin and its corresponding glucuronides or methylated derivative isorhamnetin are bioactive [8]. The antioxidant capacity and modulatory effects on oxidative stress biomarkers and inflammatory mediators were studied using Caco-2/TC7 cells (human colon carcinoma), which are one of the *in vitro* systems most largely used to mimic the intestinal mucosa. Despite the neoplastic origin, these cells have the capacity to spontaneously differentiate into mature enterocytes and to express brush border enzymes. Neutral Red uptake assay on Caco-2 cells demonstrated the absence of cytotoxicity for a wide range of concentrations of CP. The immune-modulatory and antioxidant effect of CP extracts was proven by *in vitro* and *in vivo* assays [51, 52]. It might be associated with the presence of various compounds such as favonols and aglycone derivatives, phenolic acids and derivatives [8, 48]. Extracts from fermented CP markedly inhibited the inflammatory status of Caco-2/TC7 cells, as induced by treatments with TNFα, IL-1β and IFN-γ. Fermented CP also contributed to maintain the integrity of the tight junctions, even if subjected to negative stimulation, and markedly inhibited the synthesis of IL-8 and TFNα, after treatment with IFN-γ. Natural polyphenolic extracts, especially flavonoids, modulated the inflammation of Caco-2 cells, which were stimulated by cytokines and chemokines (e.g., IL-1, IL-6, IL-8 and TNF-α) [53]. The inhibition of these pathways at any point of the cascade repressed the synthesis of these proteins and/or of their reaction products [54]. TNFα is a pleiotropic inflammatory cytokine, which mediates inflammation, immune-response and apoptosis [55]. A large spectrum of diseases involved the over production or the persistent activation of TNFα [56]. Low levels of TNFα contribute to homeostasis by regulating the body circadian rhythm [57]. Fermentation of CP, especially with *L*. *brevis* POM4 and *L*. *rossiae* 2LC8, markedly affected the level of TNFα by Caco-2 cells. The antioxidant effect on cultured cells was higher than that found with extracts from CP and CP-CT. The protective effect was investigated through the determination of intracellular ROS and detoxification by DCFH-DA assay. Also in this case, ESE from fermented CP, mainly with *L*. *plantarum* CIL6 and *L*. *brevis* POM4, showed a markedly higher antioxidant activity on Caco-2 cells than ESE from CP-CT. Besides, the concentrations of prostaglandins PGE2, small-molecule derivatives of arachidonic acid produced by cyclooxygenases, and PG synthases were positively affected by fermented CP. PGE2 are mediators of active inflammation by promoting local vasodilatation, attraction and regulation of multiple functions of different immune cells. As expected, the incubation of Caco-2 cells with IL-1β resulted in an increase in PGE2 synthesis [58], whereas the treatment with extracts from fermented CP inhibited the synthesis of PGE2 *in vitro*.

## Conclusions

This study falls within the framework of the industrial exploitation of prickly pear by-products. Lactic acid fermentation, one of the oldest and most low‐input biotechnologies used in food bio‐preservation, could be a valuable and innovative strategy to exploit the intrinsic features of *Opuntia ficus-indica* L. cladodes. The mechanisms by which selected lactic acid bacteria fulfil the role of efficient cell factories to synthesize functional biomolecules from *O*. *ficus-indica* L. cladodes was hypothesized. Two flavonoid derivatives (kaemferol and isorhamnetin) were identified in the ethyl acetate extracts, which were considered to be the major compounds responsible for the increased radical scavenging activity and immune-modulation features. Flavonoid metabolites act at multiple levels, through the inhibition of IL-8 and TFNα pathways, decreasing the production of free radicals, suppressing the activity of COX- 2 and inhibiting the synthesis of PGE2. With the perspective of producing a functional ingredient, dietary supplement or pharmaceutical preparation, fermented CP extract may perform an anti-inflammatory adjuvant to restore homeostasis under conditions of cellular stress.

PermutMatrix analysis based on compositional (vitamin C, total carotenoids, GABA, kaemferol, and isorhamnetin), and functional (antioxidant activity, nitric oxide release, TNFα, ROS, TEER, PGE2, IL-8 levels) data showed that all fermented cladode pulp, especially those fermented with *L*. *plantarum* strains and *L*. *brevis* POM4, enhanced the antioxidant and immune-modulation features of cladode pulp (Fig 7).

## Supporting Information

S1 Fig DPPH radical scavenging activity of crude and enzimatically digested water-soluble extracts (WSE). DPPH radical scavenging activity of crude (black bars) and enzimatically digested (white bars) water-soluble extracts (WSE) from cladode pulp (CP) without bacterial inoculum and chemically acidified (CP-CT), and CP fermented with *Lactobacillus plantarum* CIL6 (CIL6) and 1MR20 (1MR20), *Lactobacillus brevis* POM4 (POM4), *Lactobacillus rossiae* 2LC8 (2LC8) and *Pediococcus pentosaceus* CILSWE5 (CILSWE5). Butylatedhydroxytoluene (BHT) was used as positive control. (± SD) of three independent experiments performed in triplicate. Bars with different superscript letters differ significantly (P<0.05).
(DOCX)
