# Correction: Lactic Acid Fermentation of Cactus Cladodes (*Opuntia ficus-indica* L.) Generates Flavonoid Derivatives with Antioxidant and Anti-Inflammatory Properties

**DOI:** 10.1371/journal.pone.0155156

**Published:** 2016-05-03

**Authors:** Pasquale Filannino, Ivana Cavoski, Nadia Thligene, Olimpia Vincentini, Maria De Angelis, Marco Silano, Marco Gobbetti, Raffaella Di Cagno

The third author’s name is spelled incorrectly. The correct name is: Nadia Thligene. The correct citation is: Filannino P, Cavoski I, Thligene N, Vincentini O, De Angelis M, Silano M, et al. (2016) Lactic Acid Fermentation of Cactus Cladodes (*Opuntia ficus-indica* L.) Generates Flavonoid Derivatives with Antioxidant and Anti-Inflammatory Properties. PLoS ONE 11(3): e0152575. doi:10.1371/journal.pone.0152575
