# Supplementary material for: Lactic Acid Fermentation of Cactus Cladodes (Opuntia ficus-indica L.) Generates Flavonoid Derivatives with Antioxidant and Anti-Inflammatory Properties
Source: PLoS One. 2016 Mar 29;11(3):e0152575. doi: 10.1371/journal.pone.0152575 (PMC4811579; doi:10.1371/journal.pone.0152575)
Supplement: S1 Fig — DPPH radical scavenging activity of crude (black bars) and enzimatically digested (white bars) water-soluble extracts (WSE) from cladode pulp (CP) without bacterial inoculum and chemically acidified (CP-CT), and CP fermented with Lactobacillus plantarum CIL6 (CIL6) and 1MR20 (1MR20), Lactobacillus brevis POM4 (POM4), Lactobacillus rossiae 2LC8 (2LC8) and Pediococcus pentosaceus CILSWE5 (CILSWE5). Butylatedhydroxytoluene (BHT) was used as positive control. (± SD) of three independent experiments performed in triplicate. Bars with different superscript letters differ significantly (P<0.05). (DOCX) [file pone.0152575.s001.docx]

**S1 Fig. DPPH radical scavenging activity of crude and enzimatically digested water-soluble extracts (WSE).** DPPH radical scavenging activity of crude (black bars) and enzimatically digested (white bars) water-soluble extracts (WSE) from cladode pulp (CP) without bacterial inoculum and chemically acidified (CP-CT), and CP fermented with *Lactobacillus plantarum* CIL6 (CIL6) and 1MR20 (1MR20), *Lactobacillus brevis* POM4 (POM4), *Lactobacillus rossiae* 2LC8 (2LC8) and *Pediococcus pentosaceus* CILSWE5 (CILSWE5). Butylatedhydroxytoluene (BHT) was used as positive control. (± SD) of three independent experiments performed in triplicate. Bars with different superscript letters differ significantly (P<0.05).
